# Revolutionizing pediatric orthopedics: GPT-4, a groundbreaking innovation or just a fleeting trend?

**DOI:** 10.1097/JS9.0000000000000610

**Published:** 2023-09-21

**Authors:** Shaoting Luo, Linfang Deng, Yufan Chen, Weizheng Zhou, Federico Canavese, Lianyong Li

**Affiliations:** a Department of Pediatric Orthopedics, Shengjing Hospital of China Medical University, Shenyang; b Department of Nursing, Jinzhou Medical University, Jinzhou, Liaoning, People’s Republic of China; c Department of Pediatric Orthopedic Surgery, Lille University Centre, Jeanne de Flandre Hospital, Lille, France

*Dear Editor*,

Pediatric orthopedics is dedicated to the diagnosis and treatment of musculoskeletal problems in children and adolescents. It deals with a broad spectrum of conditions, such as congenital disorders, growth-related issues, and tumors of the skeletal and muscular system. It differs from adult orthopedics because this field caters to growing patients, necessitating unique surgical approaches and postoperative care^1^.

China, however, has a critical shortage of pediatric orthopedic surgeons, leaving the vast majority of the extensive pediatric population to be managed by general orthopedic surgeons. This means many children lack the specialized treatment they require, potentially resulting in permanent physical damage^2^. A potential solution to this problem may lie in the emerging field of artificial intelligence (AI), particularly with the language-understanding capabilities of ChatGPT, developed by OpenAI^3^.

ChatGPT, an advanced natural language processing model, is adept at facilitating fluent conversations, retrieving information, and answering questions in a professional way. The most recent iteration, GPT-4, not only builds upon these capabilities but also adds image comprehension to its repertoire, making it even more applicable across various fields, including healthcare^4,5^.

Despite the acclaim GPT-4 has received in areas such as neurology^6^, pediatrics^7^, oncology^8^, and pathology, its potential in pediatric orthopedics remains largely uncharted. Our study proposes to use an online survey to examine GPT-4’s possible contributions to pediatric orthopedics. In this survey, GPT-4 assumes various roles across all stages of treatment. A panel of professional pediatric orthopedic surgeons will assess the accuracy and adaptability of GPT-4’s decisions, enabling a comparison of GPT-4’s performance against that of human physicians in pediatric orthopedics (Fig. 1).

**Figure 1:**
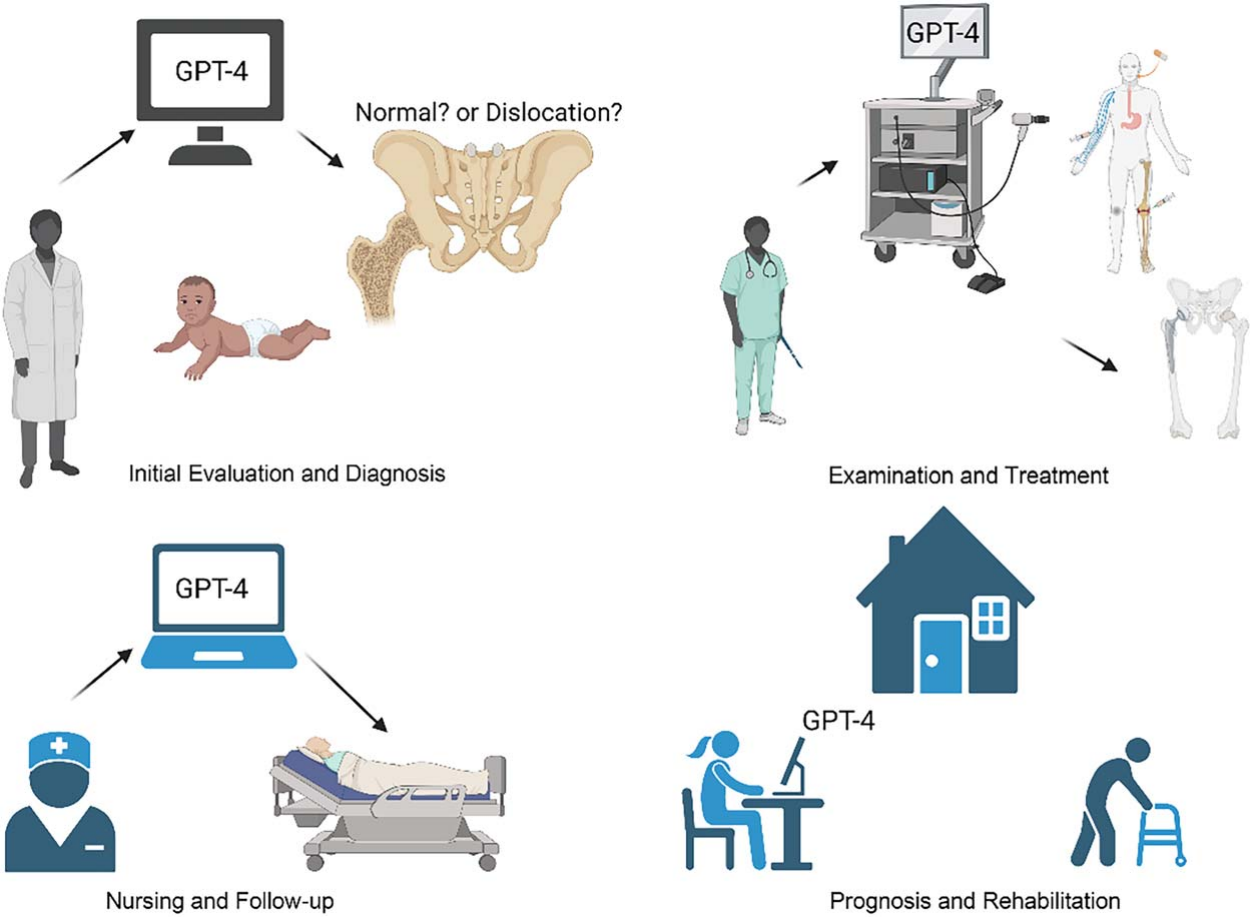
The prospective uses for GPT-4 in various facets of pediatric orthopedics (created with Biorender.com).

## How can GPT-4 help pediatric orthopedic surgeons in initial evaluation and diagnosis?

GPT-4 presents a valuable tool for aiding orthopedic surgeons in the initial stages of evaluation and diagnosis. This includes areas such as symptom analysis, medical history review, physical examination guidance, disease prediction, research assistance, and improved communication. These capabilities were put to the test using a simulated case of developmental dysplasia of the hip (DDH), a condition commonly encountered in pediatric orthopedics. DDH, which affects approximately 1 in 1000 births, is associated with multiple risk factors. In our study, we created two scenarios incorporating various risk factors for DDH. GPT-4 demonstrated its effectiveness by accurately determining the likelihood of DDH and conveying the diagnosis professionally to the simulated patient’s parents (Supplementary Figure 1, Supplemental Digital Content 1, http://links.lww.com/JS9/B28). Despite these positive results, GPT-4 did exhibit limitations in diagnosing conditions that were asymptomatic or had elusive symptoms, such as subacute osteomyelitis. While it shows proficiency in identifying diseases presenting typical symptoms, GPT-4’s accuracy diminishes when dealing with ambiguous symptomatology (Supplementary Figure 2, Supplemental Digital Content 1, http://links.lww.com/JS9/B28). As such, GPT-4 should not be seen as a substitute for the expert knowledge and clinical judgment of professional pediatric orthopedic physicians. While GPT-4 holds promise in enhancing efficiency and accuracy in assessment and diagnosis, it is important to underscore that final medical decisions should remain the domain of healthcare professionals. GPT-4 is best utilized as an auxiliary tool to assist in disease assessment and to provide potential diagnostic suggestions. Given the complexity and diversity of clinical diagnoses, the optimization and improvement of GPT-4’s role in clinical diagnostics is an ongoing endeavor.

## How can GPT-4 help pediatric orthopedic surgeons in examination and treatment?

GPT-4 possesses extensive potential for pediatric orthopedic examinations and treatments. It is capable of interpreting imaging and lab results, detecting abnormalities, and suggesting further tests as necessary. Additionally, GPT-4 can guide physical examinations for conditions such as DDH, considering age-related differences and providing a thorough step-by-step examination process (Supplementary Figure 3, Supplemental Digital Content 1, http://links.lww.com/JS9/B28).

In terms of treatment plans, GPT-4 can provide suggestions that align with the most recent clinical guidelines. As an example, when treating DDH, GPT-4 accurately recommends appropriate treatments for children at different developmental stages (Supplementary Figure 4, Supplemental Digital Content 1, http://links.lww.com/JS9/B28). Furthermore, the model aids in preoperative planning for complex procedures by generating a comprehensive list of required equipment, potential complications, and the sequence of surgical procedures (Supplementary Figure 5, Supplemental Digital Content 1, http://links.lww.com/JS9/B28).

GPT-4’s capability extends to patient education as well. It can effectively translate intricate medical conditions and treatments into language that is easily understandable for both patients and caregivers. This feature enhances the understanding of health conditions, augments medical knowledge, and facilitates more effective communication with healthcare teams.

In summary, GPT-4 offers significant support in pediatric orthopedic examinations and treatments. These capabilities improve the quality and efficiency of medical services, enhancing the satisfaction levels of pediatric patients and their caregivers. We eagerly anticipate further advancements of GPT-4 in the realm of pediatric orthopedic examination and treatment.

## How can GPT-4 help pediatric orthopedic surgeons in nursing and follow-up?

GPT-4 exhibits considerable promise in optimizing pediatric orthopedic postoperative care. It possesses the potential to streamline personalized pain management, elucidate the recovery process to both patients and caregivers, and supply detailed postoperative care instructions. Additionally, GPT-4 can serve as a helpful tool for caregivers in interpreting functional monitoring results and tracking the child’s recovery progress (Supplementary Figure 6, Supplemental Digital Content 1, http://links.lww.com/JS9/B28).

Considering the challenges faced in China, such as lengthy treatment periods and scarce healthcare resources, GPT-4 becomes particularly beneficial. It can develop tailored follow-up plans, issue reminders for necessary examinations, propose suitable rehabilitation exercises, and promote consistent interaction with medical professionals. Furthermore, it is capable of executing remote recovery monitoring, providing an evaluation of the child’s condition grounded on feedback from parents (Supplementary Figure 7, Supplemental Digital Content 1, http://links.lww.com/JS9/B28).

Leveraging GPT-4 for online counseling is an appreciable benefit, especially when geographical or financial hurdles present themselves. The technology can promptly address any concerns from parents, distribute educational content, and potentially detect any emerging problems early on (Supplementary Figure 8, Supplemental Digital Content 1, http://links.lww.com/JS9/B28).

To sum up, integrating GPT-4 into pediatric orthopedic postoperative care could offer substantial clinical support, alleviate stress, ease financial pressures, and enhance the overall productivity of healthcare teams. In doing so, it would pave the way for a more all-encompassing and customized care approach.

## How can GPT-4 help pediatric orthopedic surgeons in prognosis and rehabilitation?

The advent of GPT-4 may lead to significant advancements in both prognostic and rehabilitative care within the realm of pediatric orthopedics. Its capacity to process and analyze vast amounts of medical and clinical data fosters an in-depth understanding of diseases and related prognosis factors, resulting in more accurate prognostic evaluations (Supplementary Figure 9, Supplemental Digital Content 1, http://links.lww.com/JS9/B28).

By methodically analyzing an array of factors – including the type and location of the fracture, a patient’s age, sex, nutritional status, and any underlying conditions – GPT-4 can contribute to the development of highly accurate prognostic prediction models (Supplementary Figure 10, Supplemental Digital Content 1, http://links.lww.com/JS9/B28). This is of paramount importance in cases such as growth plate fractures, which could potentially influence future skeletal development, and instances where nutritional status may impact the pace of healing and overall recovery time.

The ability of GPT-4 to pinpoint negative prognostic indicators enables swift medical interventions, thereby mitigating complications in pediatric orthopedic conditions.

When it comes to rehabilitation, GPT-4’s capacity to understand individual patient conditions equips it to craft personalized rehabilitation plans. As an illustration, in patients with DDH undergoing postoperative rehabilitation (Supplementary Figure 11, Supplemental Digital Content 1, http://links.lww.com/JS9/B28), GPT-4 can assess patients at various stages of recovery and provide timely guidance on transitioning to subsequent phases. This feature is critical given that a substantial portion of pediatric orthopedic rehabilitation takes place at home.

## How can we balance the innovation and ethical issues posed by GPT-4 in pediatric orthopedics?

The integration of advanced AI models, such as GPT-4, calls for a meticulously considered strategy, given the multifaceted implications it presents.

Preserving the vital role of human judgment in healthcare is imperative. Designed to enhance and not supplant decision-making, AI models like GPT-4 should complement healthcare practices. Paramount to their application is the protection of patient autonomy and privacy, warranting informed consent before their deployment. A significant aspect of this endeavor involves offering patients the option of refusing AI-assisted care.

Given the intricacy of AI models, a high level of transparency is required in their design, utilization, and governance. This transparency could be achieved via legislation obliging AI creators to disclose the algorithmic processes and data sources behind their models. Doing so not only enhances understanding but also fosters trust between healthcare practitioners and patients.

The establishment of well-defined legal frameworks is essential to ascribe liability in instances of AI-induced errors. The responsibilities of all stakeholders involved – from AI developers and healthcare providers to end-users – must be clearly and precisely specified for the effective application of AI in healthcare. Instituting exhaustive accountability structures could mitigate potential inaccuracies and safeguard patients’ rights and interests.

Equity in accessing AI is a vital ethical issue. AI-driven healthcare solutions should not be a privilege reserved solely for affluent regions or institutions. Collaboration between policymakers and healthcare organizations is essential to ensure that underprivileged communities also enjoy the benefits of AI advancements. This may necessitate subsidizing costs or investing in the required infrastructure.

In summary, while the incorporation of AI models like GPT-4 in pediatric orthopedics entails challenges, it equally offers substantial potential advantages. If these ethical issues are effectively addressed, AI can significantly boost and advance the level of patient care. The secret resides in adhering to scientifically rigorous, unbiased, and patient-centered methodologies. Transforming these challenges into opportunities holds the potential to redefine healthcare outcomes drastically.

## Outlook

GPT-4, a cutting-edge AI technology, has substantial transformative potential in the field of pediatric orthopedics, encompassing aspects such as diagnosis, treatment, ongoing care, and prognosis. Nevertheless, several ethical and legal challenges, including data privacy, the distribution of decision-making responsibilities, and the fair allocation of technology, require attention. Therefore, developing strategies and guidelines for the ethical application of AI is critical. If these challenges can be successfully navigated, the implementation of AI could propel not only pediatric orthopedics but also the broader medical field into a promising digital era.

## Ethical approval

This study did not include any individual-level data and thus did not require any ethical approval.

## Consent

This study did not include any individual-level data and thus did not require any ethical approval.

## Sources of funding

This work was supported by the ‘Planting Plan’ Project for Clinical Research of Shengjing Hospital and the National Nature Science Foundation of China (Grant number: 81772296).

## Author contribution

S.L., F.C., and L.L.: conceived and designed the study; Y.C. and W.Z.: conceptualized the study and methodology; S.L.: wrote the manuscript; L.D.: conceptualized the study and methodology. All the authors have read and approved the final manuscript.

## Conflicts of interest disclosure

The authors declare that there are no conflicts of interest.

## Research registration unique identifying number (UIN)

Not applicable.

## Guarantor

Not applicable.

## Data availability statement

Not applicable.

## Provenance and peer review

Not commissioned, externally peer-reviewed.

## Supplementary Material
