# Supplementary material for: Revolutionizing pediatric orthopedics: GPT-4, a groundbreaking innovation or just a fleeting trend?
Source: Int J Surg. 2023 Sep 21;109(11):3694–7. doi: 10.1097/JS9.0000000000000610 (PMC10651230; doi:10.1097/JS9.0000000000000610)

**Supplementary Figure 1. Diagnostic Performance of GPT-4.** The figure illustrates the accuracy with which GPT-4 conducts diagnostics.


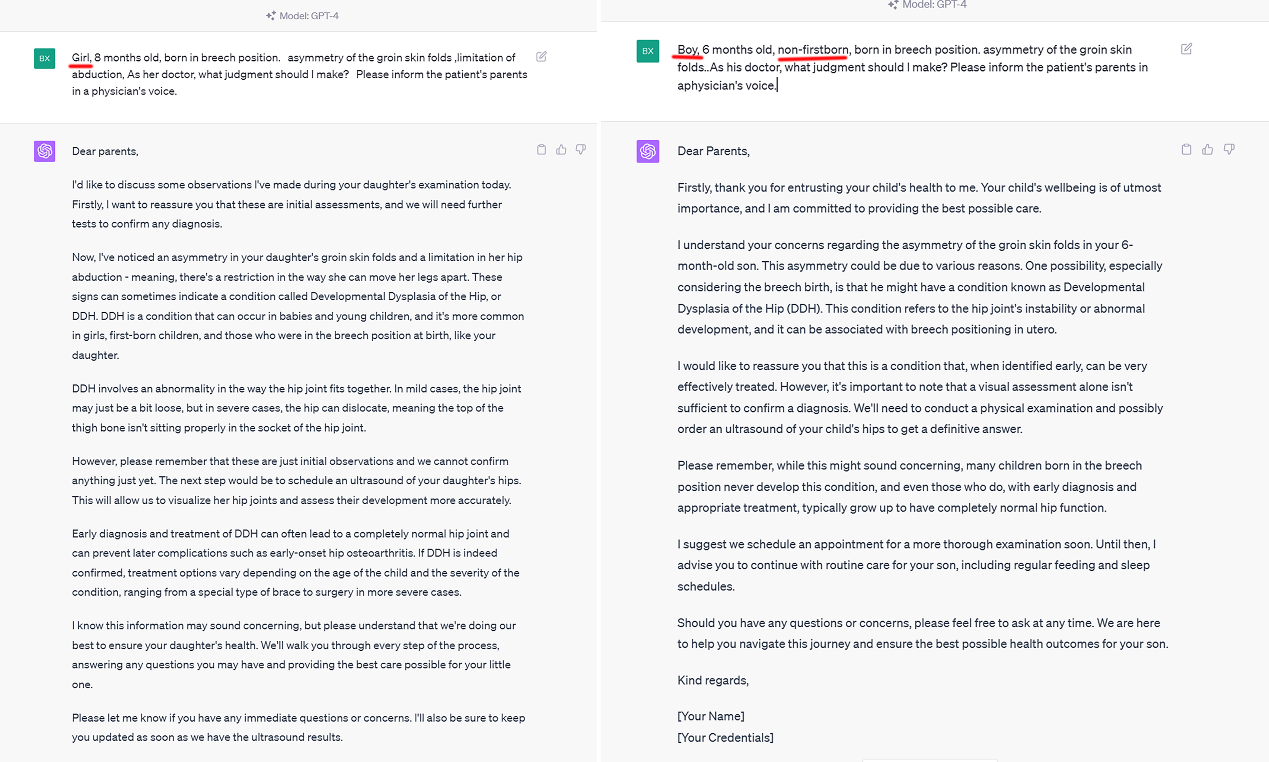


**Supplementary Figure 2. Diagnostic Accuracy of GPT-4 in Cases with Ambiguous Symptomatology.** This figure showcases the capability of GPT-4 to accurately diagnose conditions even when presented with ambiguous symptoms.


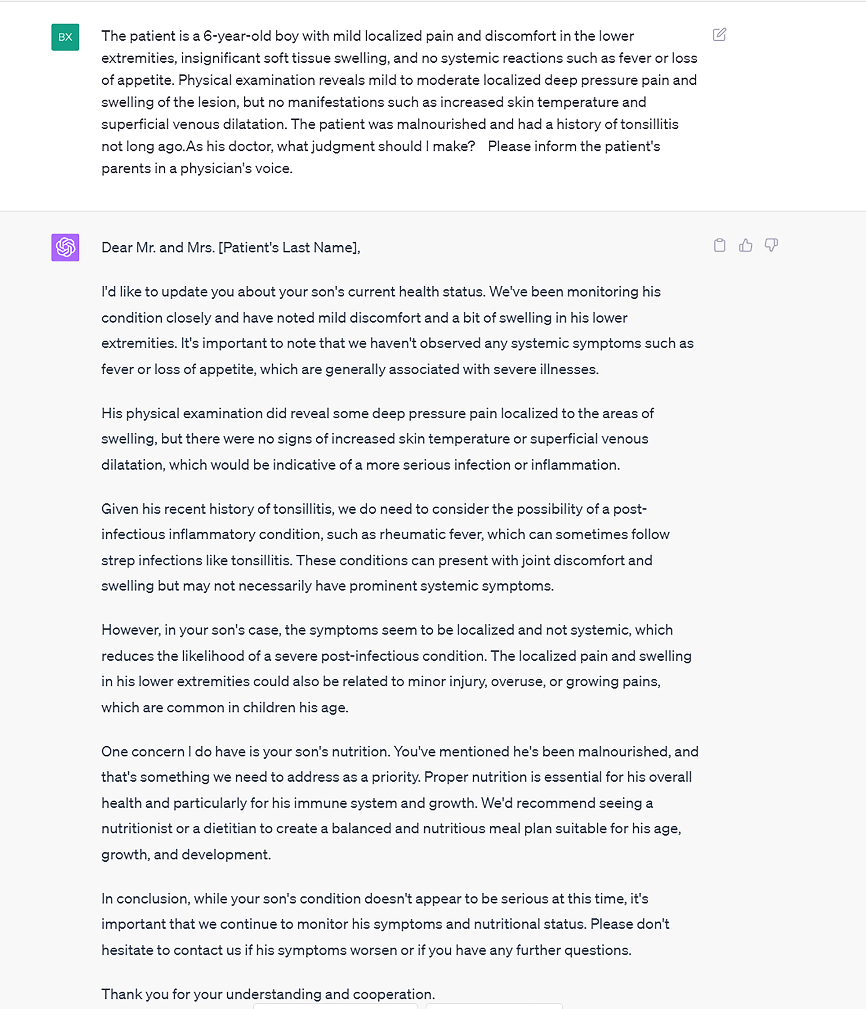


**Supplementary Figure 3. Examination Accuracy of GPT-4.** This figure illustrates how GPT-4 systematically and accurately conducts examinations, detailing each step of the process.


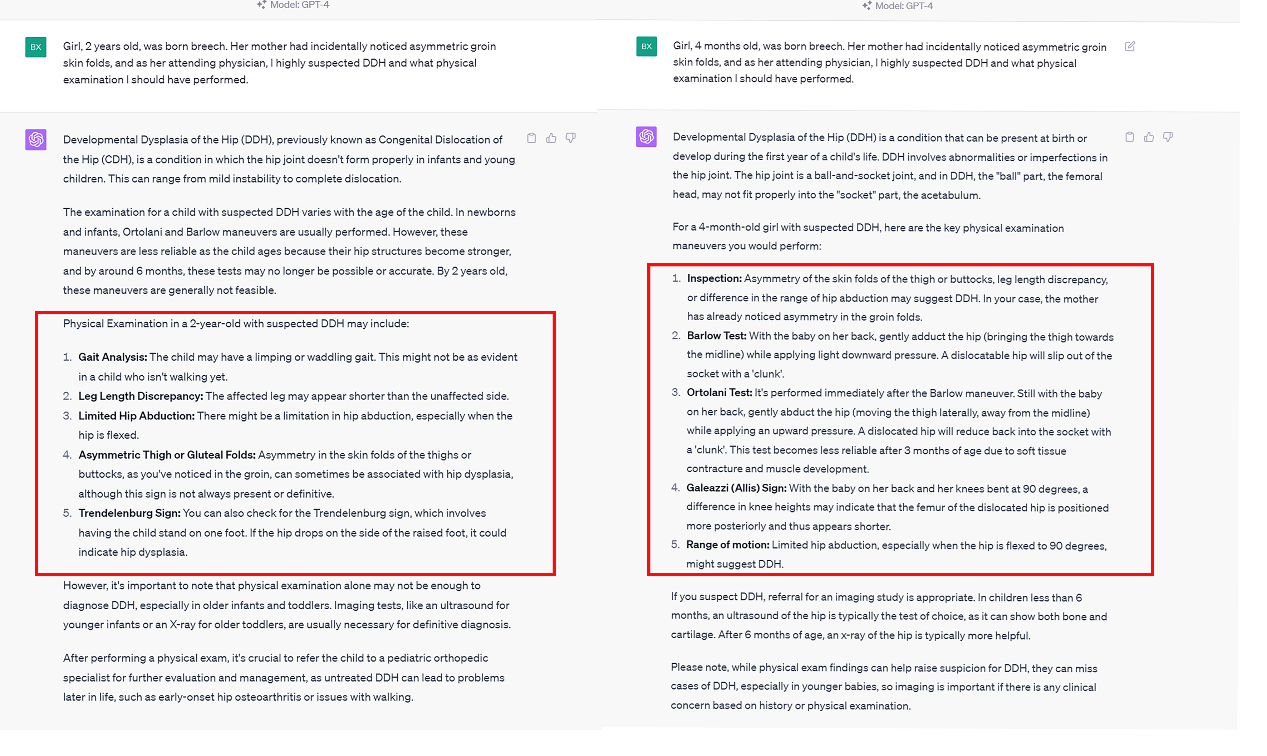


**Supplementary Figure 4. Treatment Plans Proposed by GPT-4.** This figure illustrates the capability of GPT-4 to devise appropriate treatment strategies.


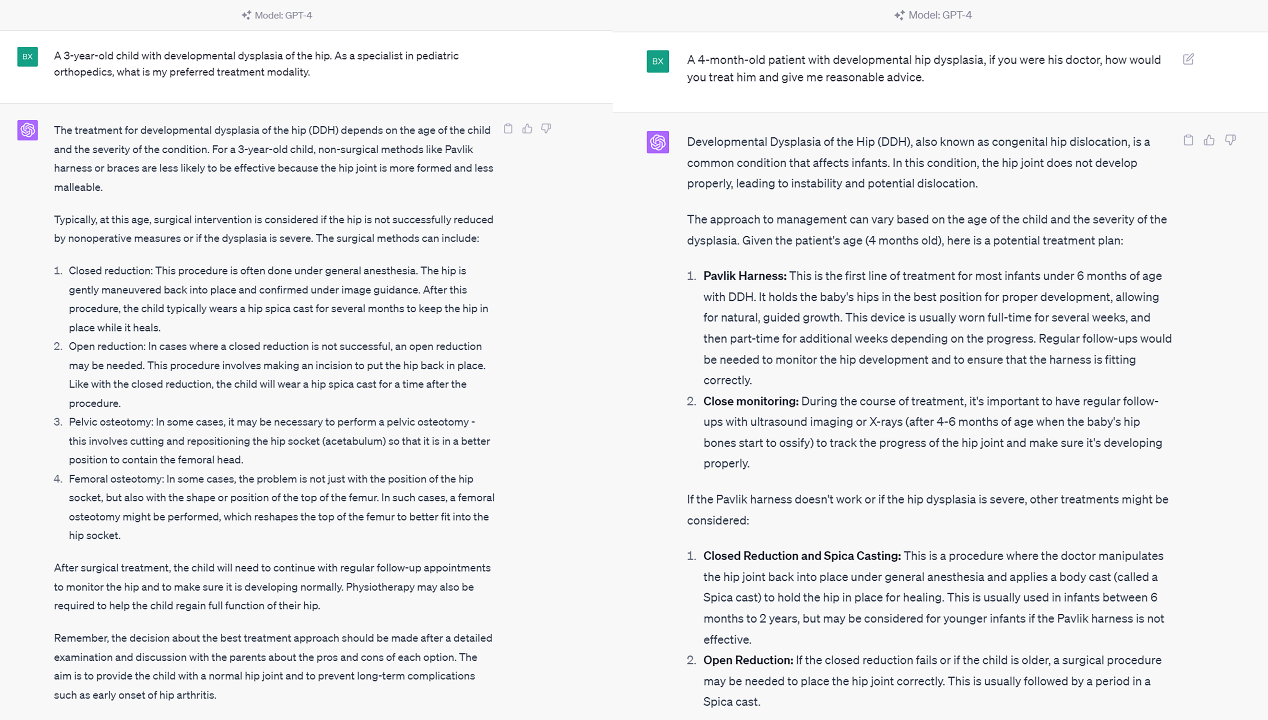


**Supplementary Figure 5. Surgical Procedures Proposed by GPT-4.** This figure highlights GPT-4's capability in preoperative planning for complex surgical procedures.


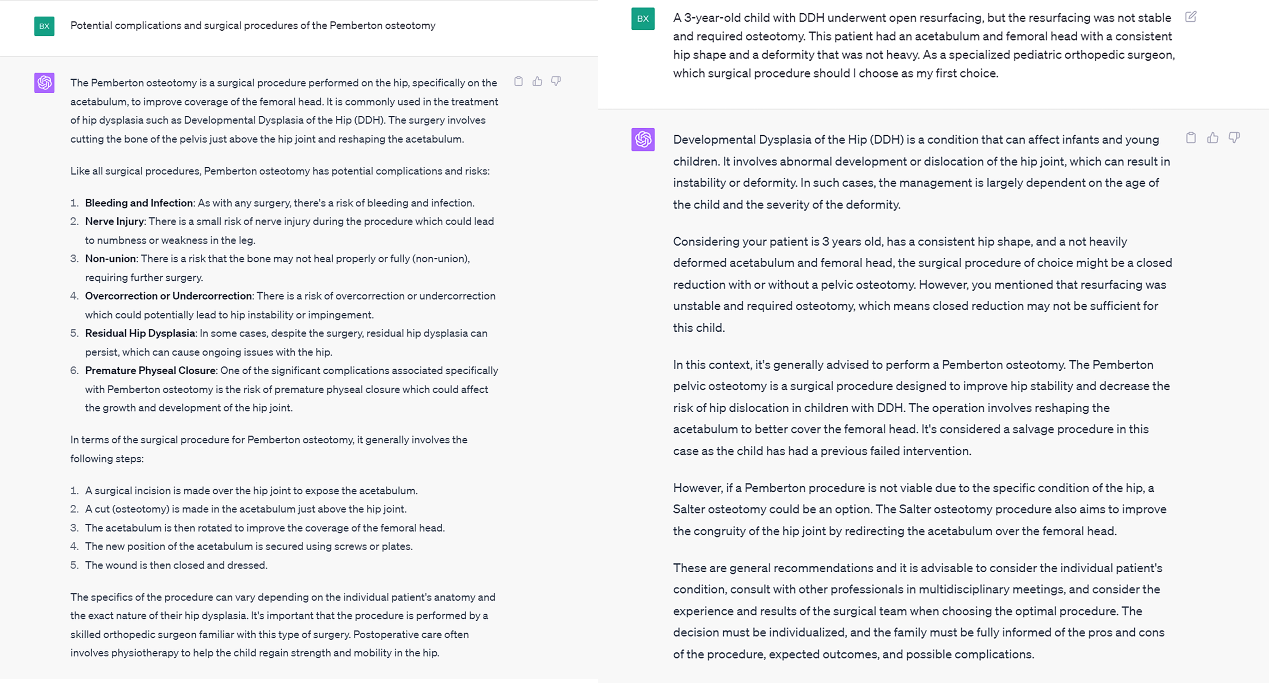


**Supplementary Figure 6. GPT-4's Interpretation of Functional Monitoring.** This figure showcases the proficiency of GPT-4 in analyzing functional monitoring results.


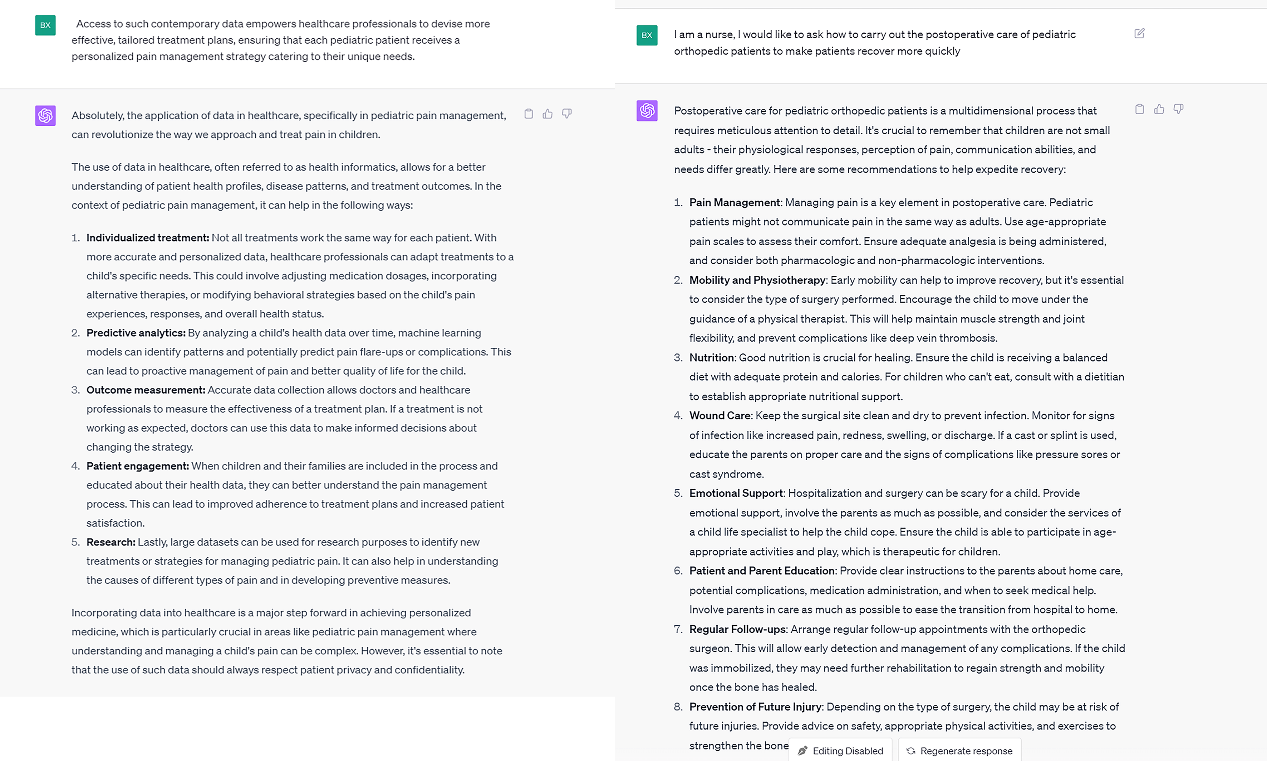


**Supplementary Figure 7. GPT-4's Evaluation of a Child's Condition.** This figure showcases GPT-4's proficiency in executing remote recovery monitoring.


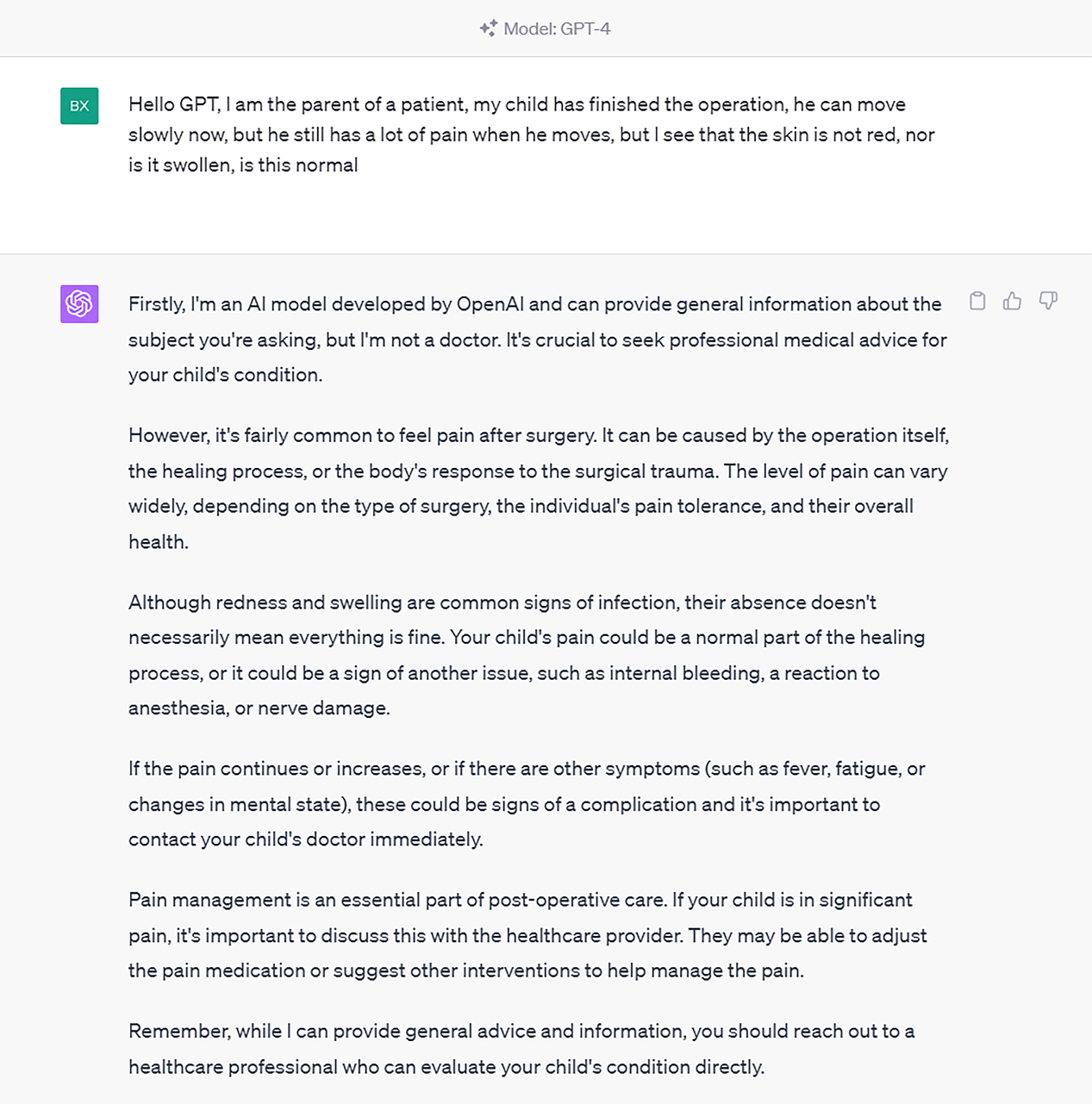


**Supplementary Figure 8. GPT-4 in Online Counseling.** This figure demonstrates how GPT-4 can proactively address and identify problems early on during counseling sessions.


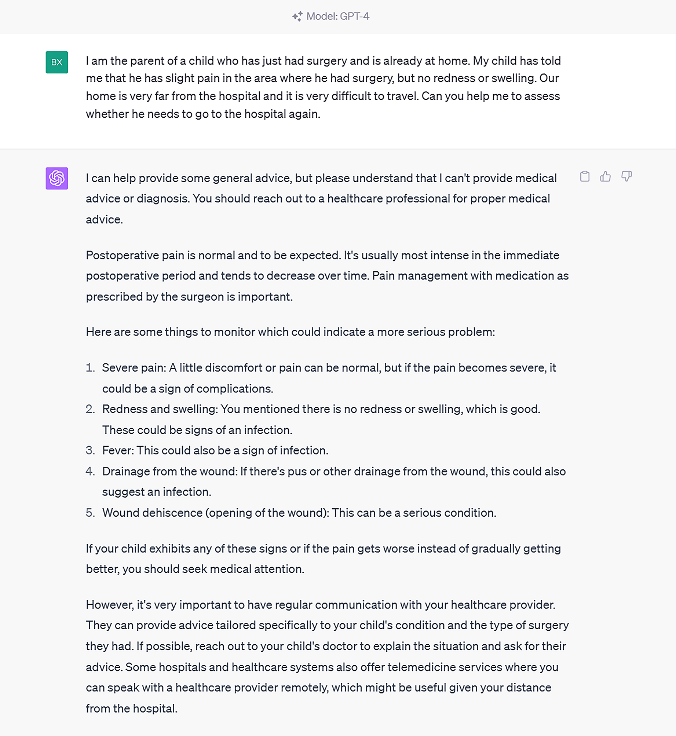


**Supplementary Figure 9. GPT-4 in Prognostication.** This figure demonstrates the capability of GPT-4 to proactively predict and analyze prognosis.


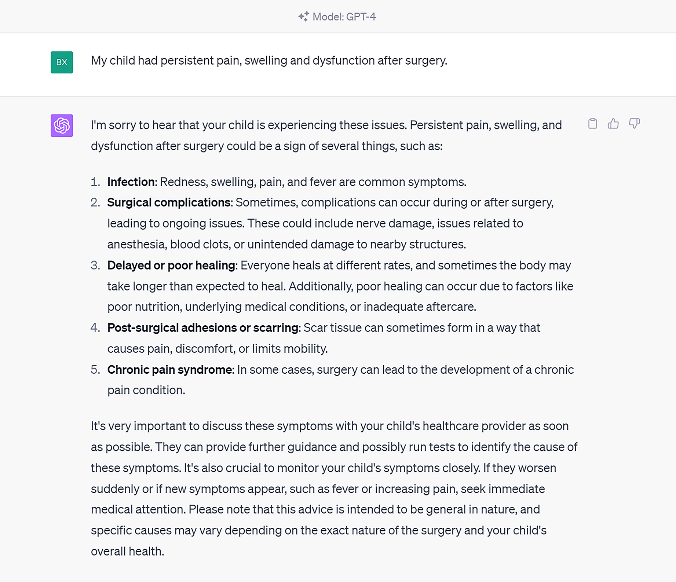


**Supplementary Figure 10. Prognostic Prediction by GPT-4.** This figure demonstrates the capability of GPT-4 to proactively predict and analyze the prognosis of underlying conditions.
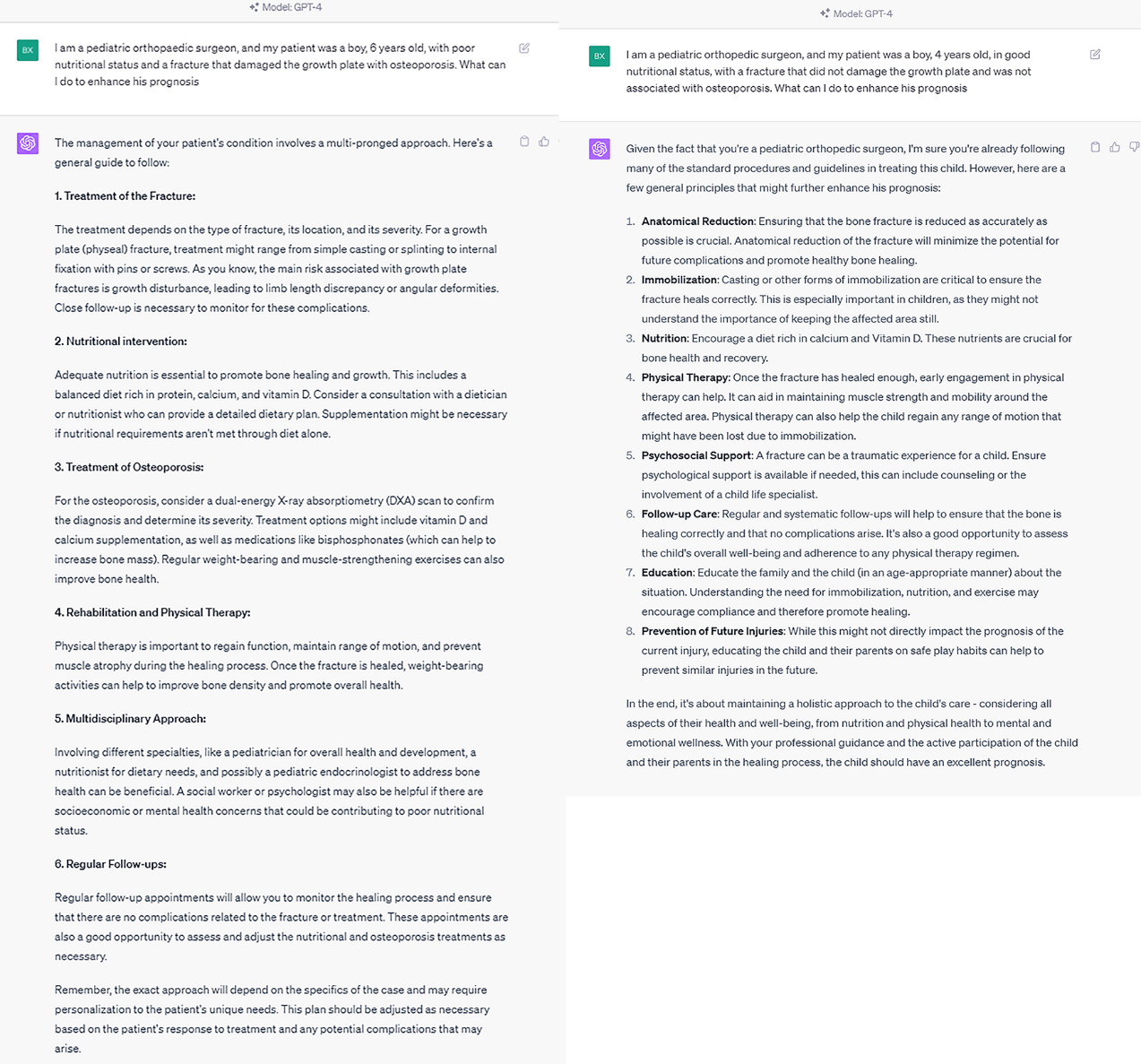


**Supplementary Figure 11. Postoperative Rehabilitation Guidance by GPT-4.** This figure showcases GPT-4's capability to offer timely and effective guidance for postoperative recovery.


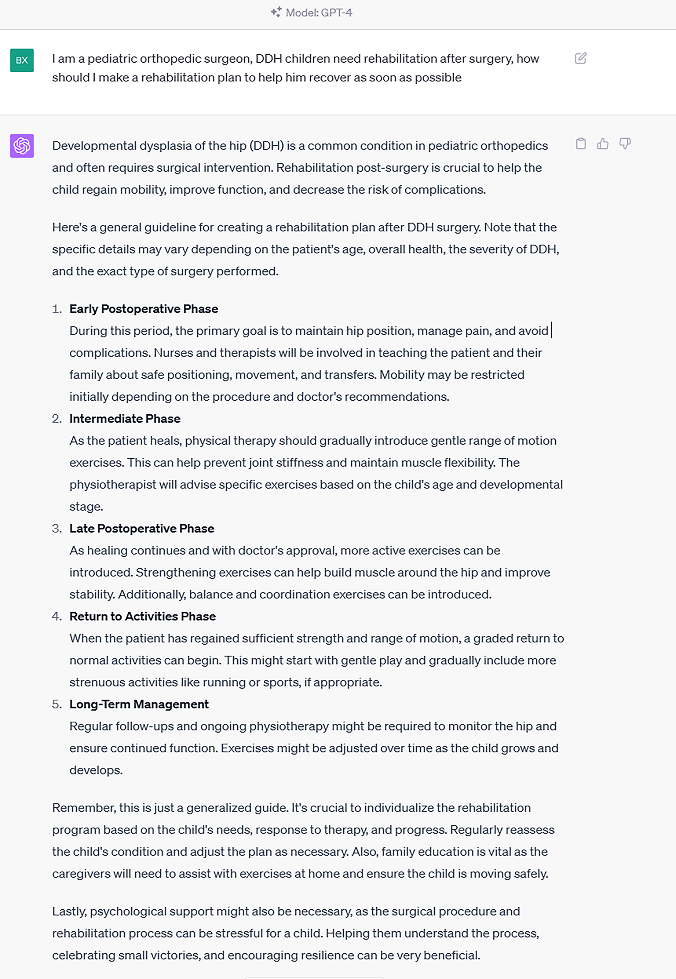

Supplement: Supplementary file 1 [file js9-109-3694-s001.docx]
